# FORGING A PATH TO DEVELOPING AGE-FRIENDLY CITIES AND COMMUNITIES IN GHANA: ISSUES AND RECOMMENDATIONS

**DOI:** 10.1093/geroni/igad104.0829

**Published:** 2023-12-21

**Authors:** Samuel Asante, Grace Karikari

**Affiliations:** Northeastern State University, Broken Arrow, Oklahoma, United States; University of North Dakota, Grand Forks, North Dakota, United States

## Abstract

The World Health Organization in 2007 launched the Age-friendly Cities and Communities (AFCC) initiative in an effort to make cities around the world more attuned to the needs of older adults. While AFCC initiatives have mostly been undertaken in developed societies around the world, developing countries appear to be excluded in this effort, as there is little to show for in the quest to creating AFCC. One of such countries is Ghana, where the proportion of older adults is expected to increase exponentially in the next decade, thereby heightening the need for AFCC. Utilizing a multiple case study approach, this paper synthesizes the procedures and findings of 16 case studies from around the world published on the eight domains of WHO’s Age-friendly Cities Framework, to make a case for developing AFCC in Ghana. Five important considerations were identified as fundamental to the effort to developing AFCC in Ghana: 1. Strengthening traditional views/perceptions on aging and older adults; 2. Involving key players in age-friendly efforts; 3. Responding to the complex interplay of needs and demands of older adults; 4. Designing policies and social programs to stimulate and enable cities and communities in Ghana to become age-friendly; 5. Developing validated instruments to assess the age-friendliness of cities and communities in Ghana. Designing and implementing AFCC require long-term commitment and devoting considerable resources to the cause. The paper concludes by discussing a culturally-appropriate model and policy recommendations to facilitate the move toward building age-friendly communities and age-inclusive cities in Ghana.

